# Assessing the effect of dynamics on the closed-loop protein-folding hypothesis

**DOI:** 10.1098/rsif.2013.0935

**Published:** 2014-02-06

**Authors:** Sree V. Chintapalli, Christopher J. R. Illingworth, Graham J. G. Upton, Sophie Sacquin-Mora, Philip J. Reeves, Hani S. Mohammedali, Christopher A. Reynolds

**Affiliations:** 1 School of Biological Sciences, University of Essex, Wivenhoe Park, Colchester CO4 3SQ, UK; 2 Department of Mathematical Sciences, University of Essex, Wivenhoe Park, Colchester CO4 3SQ, UK; 3 Laboratoire de Biochimie Théorique, CNRS UPR9080, Institut de Biologie-Physico-Chimique, 13 rue Pierre et Marie Curie, 75005 Paris, France

**Keywords:** closed loops, closed-loop hypothesis, total contact distance, connectivity, hydrophobicity, protein folding

## Abstract

The closed-loop (loop-n-lock) hypothesis of protein folding suggests that loops of about 25 residues, closed through interactions between the loop ends (locks), play an important role in protein structure. Coarse-grain elastic network simulations, and examination of loop lengths in a diverse set of proteins, each supports a bias towards loops of close to 25 residues in length between residues of high stability. Previous studies have established a correlation between total contact distance (TCD), a metric of sequence distances between contacting residues (cf. contact order), and the log-folding rate of a protein. In a set of 43 proteins, we identify an improved correlation (*r*^2^ = 0.76), when the metric is restricted to residues contacting the locks, compared to the equivalent result when all residues are considered (*r*^2^ = 0.65). This provides qualified support for the hypothesis, albeit with an increased emphasis upon the importance of a much larger set of residues surrounding the locks. Evidence of a similar-sized protein core/extended nucleus (with significant overlap) was obtained from TCD calculations in which residues were successively eliminated according to their hydrophobicity and connectivity, and from molecular dynamics simulations. Our results suggest that while folding is determined by a subset of residues that can be predicted by application of the closed-loop hypothesis, the original hypothesis is too simplistic; efficient protein folding is dependent on a considerably larger subset of residues than those involved in lock formation.

## 1. Introduction

Among the theories on protein folding, Berezovsky *et al*.'s controversial hypothesis, that the basic protein-folding unit is a closed loop (loop-n-lock) with a length of about 25–35 amino acid residues, formed by non-local hydrophobic interactions between the loop ends, is of particular interest [1,2]. This hypothesis, which builds on the non-radiative excitation energy transfer measurements of Ittah & Haas [3], is immediately attractive, as it offers the prospect of a molecular-level understanding of protein structure and folding, shedding light, for example, on the possible nature of the funnels on folding pathways. The hypothesis can be accommodated into the currently accepted mechanisms of protein folding, such as framework, hydrophobic collapse and nucleation–condensation [4–7]. Furthermore, it has potential relevance well beyond the scope of protein folding, for example in matters of protein or drug design.

Current evidence for closed loops (defined in part by a close approach in space of residues some distance apart along the polypeptide chain) comes from several observations, all of which point to a common unit of approximately 25 residues. These observations include a peak in the distribution of the length of protein chain-returns [1,2], a peak in the number of amino acid neighbours as a function of sequence distance [2], the autocorrelation function of hydrophobic residues [8], and of specific hydrophobic tripeptides [9,10], and the presence of minimally disruptive protein fragments or ‘schemas’, that can be exchanged without loss of function [11]. Once the locks have been determined (the lock is formed from residues at both ends of the loop), it is observed that hydrophobicity plots show the maximum at the lock residues [8,12] and that these lock residues tend to be conserved [13]. Elsewhere, we have shown that the closed-loop folding hypothesis is consistent with the data derived from misincorporation proton–alkyl exchange experiments and from hydrogen exchange experiments [14] that have been used to derive foldons. Thus, closed loops may provide a preferable interpretation of these exchange data because they are contiguous, unlike the foldons, which may be disjointed [15–17].

To date, support for the closed-loop hypothesis has largely been based on sequence analysis and equilibrium protein structures and has received less attention in mainstream protein-folding studies. Here, we challenge the hypothesis using results from *in vitro* protein-folding experiments and from observations of dynamic protein structures. It is known from kinetic experiments that the folding rate of a protein correlates well with total contact distance (TCD) [18]; evaluating this metric across a subset of residues, including derived lock residues and their contacts, we note a marked improvement in this correlation, suggesting that that the lock residues and their neighbours together form the folding core of the protein. To address challenges in identifying lock residues, we consider two alternative approaches for identifying this core. Methods based upon the structural and chemical properties of residues, and upon high temperature molecular dynamics (MD) simulations each produce significant overlap with the sets of locks plus contacts. We next consider evidence for loop structures of length close to 25–30 residues. Coarse-grain elastic network studies show that protein residues with high force constants (and hence greater stability) tend to have a spacing of about 24 residues, compatible with the closed-loop hypothesis. In earlier work, we found an association between sites of ligand binding and a measure of increased residue stability [19]; we here show that, across a set of diverse protein structures, loops of length of 15–30 residues are more prevalent for cases in which at least one end of the loop is in a ligand-binding site. Taken together, the results confirm that the residues predicted to mediate closed-loop formation play an important role in protein folding, but it is also reasonable to conclude that the role of the lock residues has previously been overemphasized as residues neighbouring the lock residues are also important. The scope of the hypothesis and its relevance to protein stability and to drug design is discussed.

## 2. Material and methods

### 2.1. Determination of closed loops

All loops of length of 12–50 residues with minimum heavy-atom distance of 6 Å were determined. The contact region was scored according to the number of contact neighbours, conservation and hydrophobicity [13], evaluated over a window of one, three or five adjacent residues. The highest scoring loop was determined first, and subsequent loops were identified such that there was minimum overlap between loops (but a given lock region frequently participated in two separate closed loops). The locks contained between two and eight residues, with most having four to six residues (this is the full set of lock residues). For the TCD calculations, restricted to the 43 proteins where NMR structures were available, two further refinements were made. Firstly, lock residues that did not form persistent interactions across the ensemble of NMR structures were eliminated, generally leaving a set of two to four NMR-refined lock residues. Secondly, a minimal pair of two residues was selected to form the lock based on (i) good interactions, as observed using molecular graphics and (ii) having a large number of neighbours (see the electronic supplementary material, table S1 and figure S1). The lock residues are found in all types of secondary structure, i.e. within helices, sheets, loops and β-turns, and may overlap the junction between any two such secondary structures. Further details of the method for deriving loops are given in Chintapalli *et al*. [14].

### 2.2. Distribution of loop length

In order to analyse the distribution of chain return lengths in proteins, 270 proteins, with lengths of at least 100 residues, and with well-characterized ligand-binding residues, as reported in the LPC database [20] were taken from the PDBSelect 25% list [21,22]. For each protein, all loops were found where the ends contacted to within 6 Å. The LPC database was used to identify loops for which at least one of the two loop end residues was contained within a ligand-binding site.

### 2.3. Rigidity profiles

The rigidity profiles composed of residue-by-residue force constants were calculated from the conformational fluctuations observed for a set of 98 proteins taken from the set of Yang & Bahar [23]. Each force constant characterizes the difficulty of displacing the residue in question within the overall protein structure. The proteins were represented as coarse-grain elastic networks with two to three pseudo-atoms per residue. We use an elastic network model where all the harmonic springs connecting pseudo-atoms less than 9 Å away have the same Hooke's law force constant *γ* = 0.6 kcal mol^−1^ Å^−2^. The elastic system is initially, by definition, in its equilibrium state and will undergo deformations around the equilibrium during the simulations because of the random displacement term in the Brownian dynamics equation of movement. In order to compare proteins of different sizes, the force constants were re-expressed in units of standard deviation with respect to the mean for each protein (*Z*-scores) [24,25]. An autocorrelation was carried out of both the re-expressed force constants, 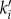, and of 99 999 sets where the re-expressed force constants were randomized within each protein. The significance of the peak in the autocorrelation was assessed by evaluating the ratio of the average value of 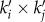 over the range 22–27 to that of the combined preceding range 15–20 and the following range 30–35. This was compared with that arising from the corresponding randomly generated values.

### 2.4. Total contact distance

The relationship between protein structure and folding rate has been well established with the observation that the log of the folding rate, ln *k_f_*, correlates with contact order, CO [26], which is defined as2.1
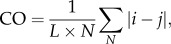
where *N* is the number of pairs of residues (*i*,*j*) that are in contact with one another, the metric |*i* − *j*| describes the separation of the residues *i* and *j* in the chain and *L* is the length of the protein. Alternatives to contact order, namely absolute contact order [27], long-range order [28,29] and TCD have been proposed. Here, we have used TCD (equation (2.2)), because TCD is insensitive as to whether immediate neighbours are included or not [18]. This makes TCD ideal for use in calculations based on selected subsets of residues and contacts. The use of CO or TCD with a subset of residues can also be justified by reference to the work on the relationship between loop length and contact order [5].2.2
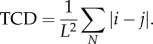
There are several sources of kinetic data for both two-state and multi-state proteins for use in such correlations [18,28,30–33], providing data for about 90 proteins. The kinetic data used in the correlation between the log-folding rate and TCD were selected largely from Zou & Ozkan [33], for the 43 unique two-state proteins where NMR structures are available (NMR structures give some indication of the fluctuations present in the protein, and hence the more important contacts within a lock). Two different sets of interactions were used in equation (2.2). These were (i) all residue–residue contacts and (ii) the interactions between the minimal pairs of lock residues plus interactions of the residues that contact the minimal pairs (other combinations are discussed in the electronic supplementary material).

In each case, a control was carried out by determining TCD for an equivalent number of contacts chosen by selecting the residues randomly; the significance of the real correlation was given by determining the proportion of cases that had a more extreme value of *r*^2^. In common with other similar studies, we have primarily omitted two intervening residues in determining |*i* − *j*| (i.e. we have excluded 1,2 and 1,3 contacts) but we have also studied the correlation between TCD and ln *k_f_* for omission of between three and 80 residues.

### 2.5. Residue property-based approach to core identification

As an alternative approach to determining the protein core, the correlation between TCD and ln *k_f_* was determined following the removal of different percentages of the residues according to their connectivity or hydrophobicity values; residues were ranked according to their connectivity or hydrophobicity and the residues with the lowest rank were removed first. The connectivity of a residue was defined as the number of other residues, at a distance of at least two residues in the chain, within a heavy-atom distance of 6 Å. Hydrophobicity was calculated according to the octanol–water partition coefficient [34]. Where multiple residues had the same rank, random sets of residues fulfilling the criteria were removed, the mean correlation for 1000 repetitions of this process being output. In order to investigate whether clusters of moderately hydrophobic residues were more important than isolated highly hydrophobic residues, residues were also removed according to the product of connectivity and hydrophobicity (where connectivity and hydrophobicity were scaled to between 0 and 1, with 1 representing the highest connectivity or hydrophobicity). We thus evaluated what percentage of residues could be eliminated while still retaining a good correlation between TCD and ln *k_f_* (a good correlation being similar to that of previous published work [18,26,28,35,36]). The optimal percentage of residues to be removed was determined by comparison with 1000 random removals at each percentage point, to generate statistics by a Monte Carlo approach. The calculations were carried out using Mathematica.

### 2.6. Molecular dynamics simulations

In order to investigate the participation of lock pairs and neighbours in initiating folding events, we analysed MD refolding simulations of four of the proteins, as described in the electronic supplementary material.

## 3. Results

### 3.1. Rigidity profile and the distribution of loop lengths

**Figure 1.**
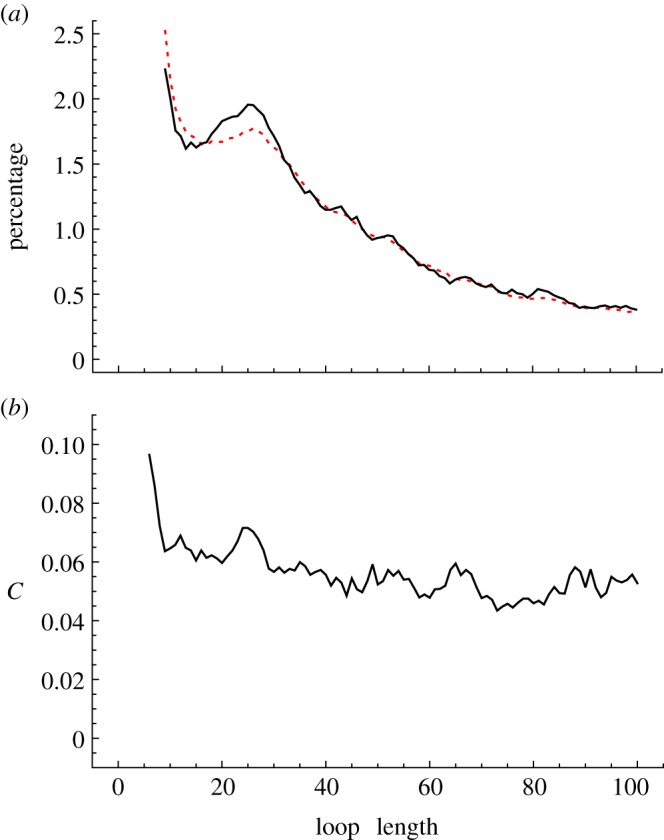
Further evidence for loops of length of approximately 25 residues. (*a*) The distribution of loop lengths in protein structures in general (dashed line) and the distribution of loop lengths where one end of the loop is in a ligand-binding site (solid line). The notable feature of the graph is the marked increase in the height of the peak at 26 (solid line), corresponding to the proposed mean length of the closed loops. The analysis is over 250 proteins from the PDBSelect25 set. Ligand-binding sites were identified from the LPC database. (*b*) Autocorrelation *C*, for reduced force constants, *k*′, greater than zero, is defined as 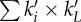, where 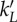 represents the value of *k*′ *L* residues further along the sequence (*C* is normalized according to the number of residues at distance *L* apart in each protein). The notable feature is the marked increase in the height of the peak at a residue separation of 24, corresponding to the proposed mean length of the closed loops.

Evidence was found to support a preference for loop lengths in protein structures of close to 25 amino acids. We examined the distribution of chain return lengths from a diverse set of 270 proteins taken from PDBSelect25 [21,22]. Considering all such chain returns, our results reproduce those of Berezovsky *et al*. [1] (dashed red line, figure 1*a*). However, where one end of the loop is part of a ligand-binding site, the distribution of loop lengths shows a more marked peak (solid black line, figure 1*a*), with the peak of this broad distribution corresponding to loops of length of approximately 26 amino acids. The relevance of this to protein folding lies in the observation that residues in the folding nucleus tend to have a high number of residue–residue contacts [37,38], while recent work has shown that this property is shared by residues in ligand-binding sites [19]. The common theme linking these two observations is entropy, because there is less loss of entropy on binding of a molecule to a rigid-binding site [19]. It therefore appears that substrate binding and protein folding tend to use the same low-entropy regions, a principle that can be exploited in drug design by tailoring drugs to bind to lock residues and the associated core residues.

If the lock residues were part of the folding nucleus, they would be expected to have a greater number of residue–residue contacts and to be held more rigidly within the protein structure. Examination of residues with a high degree of stability, as determined by coarse-grain simulations, indicated a preference for mutual separation distances of around 24 residues. Thus, the rigidity profiles were determined using Brownian dynamics simulations in which each protein was modelled as an elastic network. A force constant was then assigned to each residue according to the magnitude of the fluctuations in the protein conformation; this force constant indicates the rigidity of a residue within the overall protein structure. The autocorrelation of residues with positive reduced force constants, 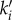 are given in figure 1*b* (negative force constants were set to zero to avoid false positives). A clear broad peak was again observed for an interresidue separation (i.e. loop length) of 22–27, giving similar results to those in figure 1*a*, and contributing to the evidence that loops of length of approximately 25 residues are significant in protein structure. Monte Carlo analysis showed the peak to be highly significant (*p* < 10^−5^).

### 3.2. Relationship between total contact distance and folding rate

**Figure 2.**
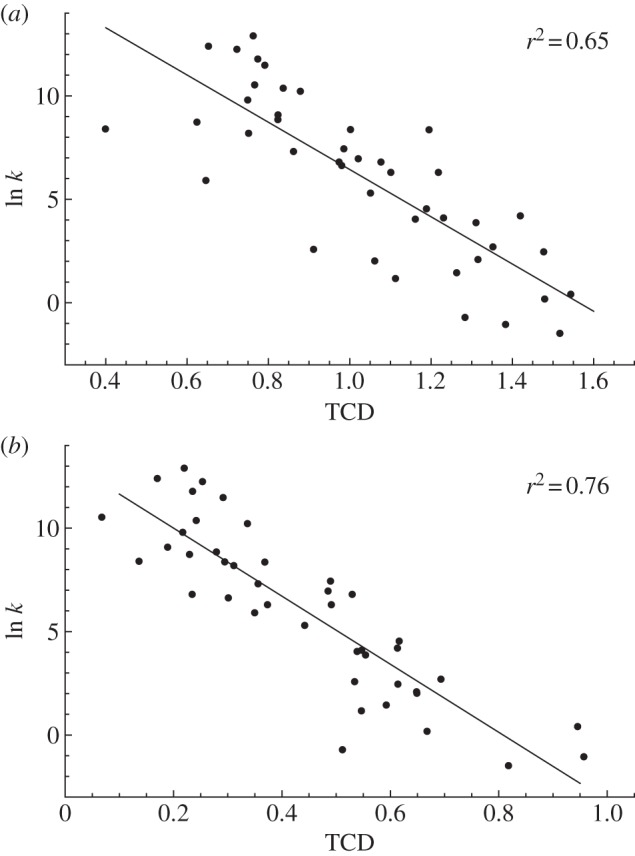
The relationship between the log of the folding rate, ln *k_f_*, and TCD for a set of 43 two-state proteins (*a*) evaluated over all residues and (*b*) evaluated over the minimal pair of lock residues plus residues that contact them. For (*a*), the outliers (for no obvious reason) are 1BA5, 1K8O, 1PSE, 1YZA, 1N88, 1PKS and 2AX5; for (*b*) the outlier is 1K8O.

We have shown that the correlation between TCD and the log-folding rate of a set of proteins, ln *k_f_*, was improved when the metric was applied to a reduced set of protein residues derived through application of the closed-loop hypothesis. In the past, much interest has been focused upon identifying specific residues that play a key role in protein folding [37,38] and upon the prediction of folding rates from protein structure [18,26–28,36]. Thus, a number of experimental studies have noted a strong correlation between the log of the protein-folding rate, ln *k_f_*, and certain protein structure-derived metrics, namely length and structural class [30,39], number of contacts [40], contact order [26,27,36], absolute contact order (ACO = CO × *L*) [27,41], ACO with corrections [39], long-range order [28] and TCD [18]. The correlation arises because both the number of contacts and the sequence distance per contact are important contributing factors to the kinetics of folding for two-state proteins, which, with some exceptions [42], have no intermediates between the denatured state and the folding state. For two-state proteins, while the folding rate correlates well with the topology (contact order), it correlates poorly with length, and so ACO does not work as well as CO, but for three-state proteins or a mixture of peptides, two-state and multi-state proteins, ACO works better than CO [27]. The correlation between ln *k_f_* and TCD for our set of 43 two-state proteins is shown in figure 2*a*. The correlation coefficient squared, *r*^2^, is 0.65, a little smaller than the value of 0.77 reported by Zhou & Zhou [18] for a similar analysis on a smaller set of 28 proteins. Our 43 proteins were selected as two-state folders and included peptides; for this relatively diverse collection neither CO nor ACO works as well as TCD (see electronic supplementary material, figures S2*a* and S3*a*). By analogy with ACO, we also tested ATCD (defined here as TCD × *L*) but this does not work as well as TCD (see electronic supplementary material, figure S4*a*).

When the evaluation of TCD was restricted to the interactions between the NMR-refined lock residues, *r*^2^ decreased considerably; reducing each lock to a minimum pair of just two residues also gave insignificant results (not shown). In these simple applications, given in the electronic supplementary material, table S2, the closed-loop hypothesis fails because the lock residues alone are insufficient to yield a good correlation.

An improved correlation was obtained by evaluating TCD across the lock residue minimum pairs and those residues in contact with them, with an *r*^2^ of 0.76 (*p* < 1 × 10^−4^) (figure 2*b*). The protein locks-and-neighbours derived core identified by this result contained a mean of 101 contacts and 30 residues per protein, representing close to 38% of all contacts and 43% of all residues (table 2; the electronic supplementary material, figure S1). Thus, although this core represents a considerably reduced subset of the total residues, performing the analysis over this core gave a substantial improvement in the correlation between TCD and the log-folding rate, suggesting that these residues play a key role in determining protein-folding rates. (ATCD, ACO and particularly CO also gave improved correlations over this reduced subset, as shown in the electronic supplementary material, figures S2*b*–S4*b*.) With just seven exceptions, the sets comprising lock residue pairs and their contacts included all of the originally identified NMR lock residues. The majority of contacts in the sets were in the same secondary structural elements as the lock residues.

Conversely, evaluation of TCD over interactions that did not include the NMR-refined lock residues or the lock pairs and their contacts resulted in insignificant correlations, as shown in the electronic supplementary material, table S2. The failure to obtain a significant correlation in the absence of the lock residues indicates that these residues contribute to protein folding.

Our results in this section concur with those of Trifonov & Berezovsky [2] regarding the prominence of loops of length of approximately 25 amino acids. However, it could be argued that this result merely reflects an artefact, for example the stiffness of the protein chain, rather than any property of locks related to protein folding. To address this issue, we monitored the variation of *r*^2^ from figure 2*a* with loop length. In these calculations, the minimum loop length used in the TCD calculations varied from two to 54 intervening residues. The results in the electronic supplementary material, figures S5 and S6 show that the correlation coefficient varies more closely with the distribution of the lengths of closed loops than with the distribution of loops in general. (Similar results, shown in the electronic supplementary material, figure S7, show that loops of length of 25–40 residues are absolutely essential for a good correlation between TCD and ln *k_f_*; but they also show that loops longer than approximately 45 residues make a significant contribution to the correlation.)

We note that, although clear criteria have been determined for identifying lock residues, and hence the core, there are nevertheless some subjective decisions involved in determining and applying these criteria. We therefore consider alternative methods for finding the core, involving structural and chemical properties of residues.

### 3.3. Identifying the core from structural and chemical properties of residues

**Figure 3.**
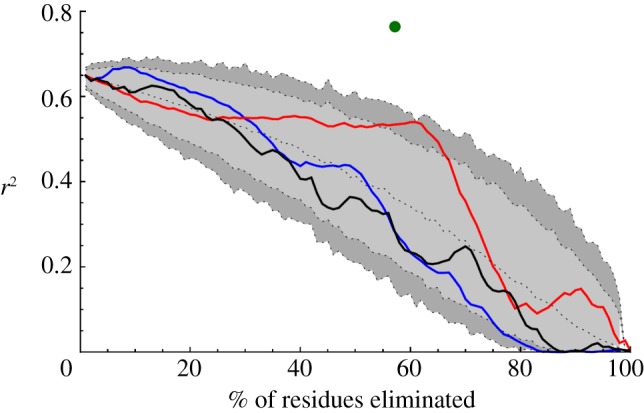
Variation of the correlation coefficient, *r*^2^, between TCD and ln *k_f_* as the percentage of residues progressively removed increases. The red, blue and black solid lines indicate the values of *r*^2^ when residues are removed according to their octanol partition coefficient, connectivity and the product of the two, respectively. Black dotted lines at increasing vertical values indicate 1%, 5%, 50%, 95% and 99% percentile *r*^2^ values, respectively, obtained by random removal of given percentages of residues from each of the set of proteins. Thus, the 95% significance region lies above the light grey area. The point corresponding to figure 2*b* is shown as a green circle. The red octanol line almost reaches 99% significance at 61% removal.

An approach based upon residue hydrophobicity identified protein cores which significantly overlapped the lock pair plus contact sets. The correlation between TCD and ln *k_f_* resulting from the removal of a given percentage of residues from each protein using a given measure (hydrophobicity and or connectivity) was calculated, and compared to statistics of the correlations arising from the random removal of the same number of residues. The removal of between 53 and 65% of residues by hydrophobicity resulted in a correlation superior to that calculated for 95% of the random sets. The first point gave a value of *r*^2^ = 0.54 while the latter gave a value of *r*^2^ = 0.46; the latter point (figure 3) was chosen, resulting in a small core of residues. Neither removal of residues according to connectivity nor the product of connectivity and hydrophobicity yielded significant correlations (figure 3); this is in line with ideas on hydrophobic collapse [7] and downplays the importance of highly connected nodes in folded proteins [37]. As in the TCD v ln *k_f_* correlations (figure 2*b* and table 1; electronic supplementary material, table S2), we required the presence of a larger set of residues that was generally about four times larger than the set of lock residues.

**Table 1.** Molecular contacts (i.e. lock residues) for acyl-coenzyme A-binding protein (pdb codes 1NTI/2ABD). The *Φ*-values are given where these are available.

1NTI	full	NMR-refined	highest *Φ*-values
loop 1	5	5	5 (0.74)
	30,31,32	30,31	32 (0.96)
loop 2	28,29,30	30	—
	73	73	73 (0.7)

The mean size of the core that remains from the hydrophobicity method (referred to as the TCD-based core; figure 3) is, at 35% of the total number of residues (see electronic supplementary material, table S3), similar to the mean proportion of locks and neighbours, of 42 ± 11%. Here, however, the core was constrained to be of a similar proportion in each protein. The residues common to the TCD-based core and the locks and neighbours of electronic supplementary material, table S1 together comprise about 58 ± 11% of the residues and are recorded in the electronic supplementary material, table S3. A comparison of the two alternative cores is difficult because they were generated using different criteria, giving rise to different sizes: the TCD core was constrained to contain 35% of the residues and was based primarily on hydrophobicity, whereas the locks' and neighbours' core varied between 24 and 65% and was determined by considering other factors in addition to hydrophobicity. Nevertheless, we assessed whether the TCD-based cores included pairs of residues for each lock (but not necessarily all the lock residues) and whether the TCD-based cores reproduced more of the lock residues than would be expected at random. The only proteins that did not satisfy either of these criteria were 1gab, 2hqi and 2jwt and for these proteins TCD-derived core neighbours could play a similar role. For some proteins, such as *1w4j* and *1ryk*, the lock residues disappeared at 57%, 62% and 64% removal, respectively, i.e. just below the 65% threshold (details are given in the electronic supplementary material, table S3).

#### 3.3.1. Molecular dynamics refolding simulations

The simulations provided no evidence that nucleation involved a small set of residues (cf. the number of lock residues) but rather that nucleation involved a larger number of residues (cf. the number of locks and neighbours). Moreover, the lock residues and their neighbours were prominent in this nucleation. For each pair of residues, the fraction of snapshots from the simulations in which the pair was in contact was calculated. This was compared to the same statistic averaged over all pairs at the same contact distance; a resulting log ratio identified pairs that were statistically more likely to form contacts than would be expected from their distance apart in the protein chain.

In acyl-coenzyme A (PDB code 1NTI), around half of the 73 contacts identified as being more likely to form contacts in the simulations (log likelihood < −0.3) were contacts between residues also observed in both the lock pairs plus neighbours and in the TCD-derived core, as indicated in the electronic supplementary material, figures S8–S10. Given that the lock pairs plus neighbours represent only 38% of contacts in the original protein, this is a significant result (*p* < 0.05; electronic supplementary material, table S3). Similar results were identified for proteins G and L (see electronic supplementary material, table S3, and figures S9 and S10). We would not expect exact agreement between the different cores as the fine details of the folding pathway may be force-field dependent [43]. In addition, *p*-values calculated for the reproduction of the lock residues are less than 0.05 for each of the three proteins.

## 4. Discussion

### 4.1. Evidence for 25mers

The loop length data and the data on the spacing of high force constant residues (figure 1) tie the observation of closed loops of around 25 residues more closely with protein folding because it associates the ends of the approximately 25mer loops with regions of high connectivity and/or rigidity, which are themselves linked with protein folding [37,38]. The study on the variation of the TCD v ln *k_f_* correlation with loop length (see electronic supplementary material, figure S7) indicates that the factor of 25 is not merely an artefact of protein structure and of peptide persistence length. Additionally, there is an interesting link between protein folding and ligand binding which is implicit in figure 1*a*. The link is implicit via the involvement of connectivity, because the folding nucleus and ligand-binding sites are associated with regions of high connectivity [19,37]. Thus, because ligands are able to bind to regions involved in stabilizing the fold, they may also in some cases assist with fold stabilization. This has been seen very powerfully in the use of tightly binding ligands to stabilize flexible structures, for example G protein-coupled receptors (GPCRs), and hence facilitate crystallization [44]. Similar principles may underlie the mechanism of pharmacological chaperones (small molecules that assist with the folding of proteins), as in the binding of SR49059 to the vasopressin receptor [45]. In both of these GPCR-based examples, the lock region and the ligand-binding region occur within the same region of the transmembrane helical bundle. Evidence for closed loops of around 25 residues is also implicit in the TCD studies (figure 2*b*) because the mean length of the closed loops in this small sample of 43 proteins is 27 residues.

As the lock residues and their neighbours are predicted to play some role in protein folding, these lock residues are given in table 1 for acyl-coenzyme A-binding protein (pdb code 1NTI), indicating that this approach is able to generate useful molecular-level information that is relevant to the folding process.

### 4.2. Total contact distance correlations

The significance of the strong correlations between TCD and the log of the experimental folding rate in figure 2*b* is twofold. Firstly, the correlation in figure 2*b* involving subsets of residues predicted to form locks gives as strong a correlation as those previously observed [18,26–28,36] even though these new correlation results are based on approximately 60% fewer residues. The improved correlation over a reduced set of residues supports the idea that protein folding is driven by a subset of residues. Secondly, because the key residues were identified through application of the closed-loop hypothesis, this suggests that the hypothesis may provide a valuable paradigm for understanding protein folding. Some support for the role of a subset of residues centred on the locks also comes from the TCD-based correlations/eliminations (figure 3; and from the MD simulations, electronic supplementary material, figures S8–S10, as these distributions were shown to overlap with the lock pairs and neighbours, electronic supplementary material, table S3). The different percentages of residues used in each method and the need to randomly eliminate residues with equal rank in the TCD-based approach may have contributed to some of the differences observed between the two alternative cores (cf. figures 2*b* and 3). In addition, the MD-based core (see electronic supplementary material, figures S8–S10) will to some extent be dependent upon the force field, even though the overall picture to emerge from the MD simulations should be reliable [43].

Some additional support for expanding the closed-loop hypothesis to include neighbouring residues also comes from *Φ*-value analysis, as discussed in the electronic supplementary material. By contrast, the closed-loop hypothesis places much emphasis on the lock residues: the weight of evidence suggests that this is somewhat simplistic.

### 4.3. The relative importance of non-lock residues

Although we set out to investigate the role of lock residues, it is very clear from the techniques applied here that knowledge of the lock residues alone does not provide a clear and sufficient description of protein folding. Several of the techniques used, as exemplified by the data in figure 2*b* (TCD v ln *k_f_* correlations), figure 3 (TCD v ln *k_f_* correlations/eliminations), electronic supplementary material, figures S8–S10 (MD simulations) and figure S11 (*Φ*-value analysis) strongly implicate a much larger protein core that is four or five times larger than the set of lock residues, but that is nevertheless much smaller than the set of all residues. This protein core has similarities to the concept of the extended nucleus described by Fersht [5]. The overlap between the locks and neighbours, the TCD-based core (figure 3) and the core derived from the MD simulations (see electronic supplementary material, figures S8–S10) indicates a common set of important residues that includes the lock residues.

Thus from this work and previous studies [5], it seems that the lock residues are not the only residues with near-native structure in the transition structure for folding or comprising the protein core. For example, in the WW domain (code 1E0L), the largest *Φ*-values are for residues in β-turns (residues 14, 15 and 26), which are not part of the locks. These two β-turns are strategically placed to facilitate the folding of loops 8–22 and 20–36, which we have identified as closed loops. Thus in 1E0L, the β-turn may be more sensitive to substitution than the locks with regard to the formation of the closed loop, especially as the loop closure is not driven by a single residue. However, in the MD simulations on 1E0L, β-turns residues 14, 15 and 16 make a few short-range contacts, whereas lock residues 8, 20, 22 and 36 make significant long-range contacts, results not shown. A similar effect is observed in protein G (pdb code 3GB1) [46], protein L (pdb code 2PTL) [47] and phosphotransferase (pdb code 1FYN) [48]. Thus, despite a slight preference for lock residues to have high median *Φ*-values (see electronic supplementary material, table S6), a high *Φ*-value does not necessarily imply a key role in nucleation [48,49]. In other proteins, high *Φ*-values are recorded for residues in the vicinity of the locks, particularly those in the same secondary structural element as the lock, indicating that for the lock to form, the secondary structure in which it resides may also have to form [6,50,51]. A similar conclusion was drawn from a re-evaluation of the native-state hydrogen exchange experiments in the light of the closed-loop hypothesis [14].

Rustad & Ghosh [39] found that absolute contact order could be significantly improved by explicit corrections for nested (this could include omega loops [52]) or linked (i.e. overlapping) loops. While such loops do not feature significantly in the original closed-loop hypothesis, as formulated by Berezovsky *et al*. as the closed loops should be non-overlapping (bar a small overlap of about five residues), we find that a reasonable number of nested and linked loops are taken into account in our approach via the lock-neighbours, as illustrated in the electronic supplementary material, figure S12 for protein 2rpn, a yeast SH3 domain; this may be one reason why it is necessary to supplement the locks with their neighbours.

In a similar vein, local contacts have no formal place within the closed-loop hypothesis, and indeed non-local contacts are known to dominate the barrier-crossing process [33]. Local contacts nevertheless play a role in folding [33,53] and the electronic supplementary material, figure S7 shows that ‘local TCD’ correlates with ln *k_f_* (a similar correlation with ‘local contact order’ was shown by Zou & Ozkan [33]). Indeed local contacts have been discussed above with regard to high *Φ*-values and native-state hydrogen exchange experiments. The inclusion of lock-neighbours automatically introduces a number of local contacts into TCD or related metrics, at least in the vicinity of the locks, as shown by the neighbouring circles in the electronic supplementary material, figure S12. Thus, our modification of the closed-loop hypothesis includes local interactions, overlapping loops and nested loops that interact with the lock residues, possibly giving increased prominence to the lock residues.

Elsewhere we have observed that it is not yet possible to determine the lock residues precisely, because different authors using slightly different methods may only agree to within one or two residues [14]. Electronic supplementary material, table S6 indicates that *Φ*-values may offer an indication as to whether residues participate in lock regions but the data are far from definitive. Thus, although we have identified a significant set of residues, the precise functional distinction between lock residues and non-lock residues is not clear at the present time. Based on the current TCD calculations, for some dynamic applications it may therefore be more useful to distinguish between core (cf. extended nucleus) and non-core residues. Here, the core could be derived from the lock pairs and neighbours [14], from the elimination of residues during TCD-based simulations (figure 3) or from MD simulations. For some proteins, for example 2jwt, the locks' and neighbours' core is a relatively low percentage (24%) of the total number of residues (13 residues out of a total of 54), whereas for others (e.g. 1wiu), it is a relatively high percentage (65%), as shown in table 2.

**Table 2.** The extent of the protein core as defined by residues neighbouring the lock residues. The table shows both the number of contacts and the number of residues involved in the correlations given in figure 1 (cf. electronic supplementary material, figure S1).

	full set of contacts (cf. figure 2*a*)		lock pairs + contacts (cf. figure 2*b*)		
protein	residues	contacts	residues	contacts	ln *k_f_*
1aey	58	221	31	110	2.09
1aps	98	426	53	213	−1.48
1ba5	53	171	22	65	5.91
1cis	66	272	33	128	3.87
1e0l	37	94	11	17	10.37
1e0m	37	101	13	29	8.85
1fex	59	207	22	58	8.19
1fkr	107	424	51	186	1.45
1g6p	66	260	21	59	6.30
1gab	53	189	17	44	12.7
1hdn	85	369	44	153	2.70
1idz	54	173	15	39	8.73
1imp	86	343	39	139	7.31
1k0s	151	613	63	250	7.44
1k8o	87	324	41	125	−0.71
1k9q	40	114	12	28	8.37
1l2y	20	42	6	7	12.4
1n88	96	398	51	219	2.02
1nti	86	364	35	139	6.96
1nyg	58	212	27	83	4.54
1o6x	81	271	22	54	6.63
1pba	81	293	23	65	6.80
1pks	76	325	46	191	−1.05
1pse	69	233	32	97	1.17
1rfa	78	307	27	85	8.36
1ryk	69	280	17	43	9.08
1srm	56	192	28	79	4.04
1ss1	60	234	21	65	11.48
1w4e	45	159	18	56	10.22
1w4j	51	170	18	53	12.25
1wiu	93	408	60	244	0.41
1yza	106	352	28	76	8.40
2ait	74	306	34	125	4.20
2ax5	99	350	51	157	2.58
2bth	45	146	15	39	11.78
2hqi	72	342	39	144	0.18
2jwt	54	191	13	16	10.53
2pdd	43	135	14	31	9.80
2ptl	62	245	36	108	4.10
2rpn	58	250	25	88	2.46
2vil	126	559	66	293	6.80
3gb1	56	201	23	72	6.30
3mef	69	242	24	73	5.30

There are long-range contributions to folding. Electronic supplementary material, figure S7 shows that loops as long as 80 residues make a significant contribution to the correlation shown in figure 2*a*. This is consistent with the observations that locks from different closed loops tend to cluster together [14]. Thus, while the closed-loop hypothesis is important for determining the lock residues, the folding process is certainly not local to the interactions within the closed loop.

### 4.4. Protein stability

**Figure 4.**
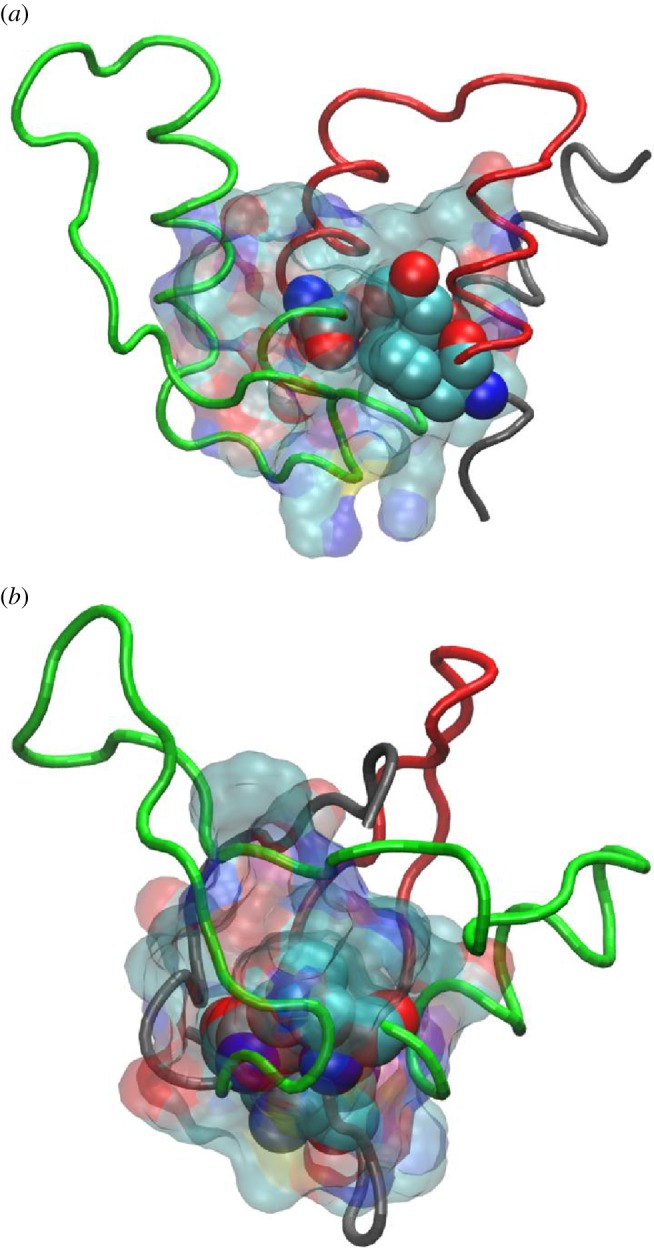
The protein cores for (*a*) 1nti and (*b*) 1rfa, as determined from the minimal pairs of lock residues (opaque, spacefill) and their equally important contacts (transparent, spacefill). The first closed loop is coloured red, the second green.

As there is much interest in modifying protein stability, e.g. as an aid to crystallization [44,54], the concept of identifying the protein core from the closed loops could be very useful, (i) for ensuring that the core is maintained under mutagenesis and (ii) for identifying areas of low stability (e.g. non-core regions) where an increase in stability could be most beneficial. Increases in stability can be engineered through mutation [54–56] or can come from a molecular chaperone binding to the lock residues/protein core [45]. The protein cores for acyl-coenzyme A-binding protein and the Ras-binding domain of c-Raf-1 (PDB codes 1NTI and 1RFA, respectively) are shown in figure 4; the cores for the 43 proteins are shown in the electronic supplementary material, figure S1.

## 5. Conclusion

We find additional new evidence for the importance of approximately 25mer closed loops from both the autocorrelation of residues with high force constants, as determined by the elastic networks, and from the distribution of loop lengths where one end of the loop is part of the ligand-binding site. However, we find that the closed-loop hypothesis is somewhat lacking in that the locks themselves are certainly not sufficient for obtaining a good correlation, but rather that the locks need to be supplemented by considerably more residues. This additional requirement is not evident from analysis of structure or sequence alone but arises when the protein dynamics is considered. However, the closed-loop hypothesis may nevertheless be a useful tool for guiding experiments to determine the nature of this core, which surrounds the lock residues.

## References

[1] Berezovsky IN Grosberg AY Trifonov EN 2000 Closed loops of nearly standard size: common basic element of protein structure. FEBS Lett. 466, 283–286. (10.1016/S0014-5793(00)01091-7) 10682844

[2] Trifonov EN Berezovsky IN 2003 Evolutionary aspects of protein structure and folding. Curr. Opin. Struct. Biol. 13, 110–114. (10.1016/S0959-440X(03)00005-8) 12581667

[3] Ittah V Haas E 1995 Nonlocal interactions stabilize long range loops in the initial folding intermediates of reduced bovine pancreatic trypsin inhibitor. Biochemistry 34, 4493–4506. (10.1021/bi00013a042) 7535565

[4] Fersht AR Daggett V 2002 Protein folding and unfolding at atomic resolution. Cell 108, 573–582. (10.1016/S0092-8674(02)00620-7) 11909527

[5] Fersht AR 2000 Transition-state structure as a unifying basis in protein-folding mechanisms: contact order, chain topology, stability, and the extended nucleus mechanism. Proc. Natl Acad. Sci. USA 97, 1525–1529. (10.1073/pnas.97.4.1525) 10677494 PMC26468

[6] Gianni S Guydosh NR Khan F Caldas TD Mayor U White GW DeMarco ML Daggett V Fersht AR 2003 Unifying features in protein-folding mechanisms. Proc. Natl Acad. Sci. USA 100, 13 286–13 291. (10.1073/pnas.1835776100) PMC263785 14595026

[7] Dill KA Ozkan SB Shell MS Weikl TR 2008 The protein folding problem. Annu. Rev. Biophys. 37, 289–316. (10.1146/annurev.biophys.37.092707.153558) 18573083 PMC2443096

[8] Berezovsky IN Kirzhner VM Kirzhner A Trifonov EN 2001 Protein folding: looping from hydrophobic nuclei. Proteins 45, 346–350. (10.1002/prot.1155) 11746682

[9] Aharonovsky E Trifonov EN 2005 Protein sequence modules. J. Biomol. Struct. Dyn. 23, 237–242. (10.1080/07391102.2005.10507062) 16218751

[10] Aharonovsky E Trifonov EN 2005 Sequence structure of van der Waals locks in proteins. J. Biomol. Struct. Dyn. 22, 545–553. (10.1080/07391102.2005.10507024) 15702926

[11] Voigt CA Martinez C Wang ZG Mayo SL Arnold FH 2002 Protein building blocks preserved by recombination. Nat. Struct. Biol. 9, 553–558. 12042875 10.1038/nsb805

[12] Lamarine M Mornon JP Berezovsky N Chomilier J 2001 Distribution of tightened end fragments of globular proteins statistically matches that of topohydrophobic positions: towards an efficient punctuation of protein folding? Cell. Mol. Life Sci. 58, 492–498. (10.1007/PL00000873) 11315195 PMC11337340

[13] Yew BK Chintapalli SV Upton GG Reynolds CA 2007 Conservation of closed loops. J. Mol. Graph. Model. 26, 652–655. (10.1016/j.jmgm.2007.03.011) 17459747

[14] Chintapalli SV Yew BK Illingworth CJ Upton GJ Reeves PJ Parkes KE Snell CR Reynolds CA 2010 Closed loop folding units from structural alignments: experimental foldons revisited. J. Comput. Chem 31, 2689–2701. (10.1002/jcc.21562) 20839296

[15] Bai Y Sosnick TR Mayne L Englander SW 1995 Protein folding intermediates: native-state hydrogen exchange. Science 269, 192–197. (10.1126/science.7618079) 7618079 PMC3432310

[16] Krishna MM Lin Y Rumbley JN Englander SW 2003 Cooperative omega loops in cytochrome c: role in folding and function. J. Mol. Biol. 331, 29–36. (10.1016/S0022-2836(03)00697-1) 12875833

[17] Krishna MM Maity H Rumbley JN Lin Y Englander SW 2006 Order of steps in the cytochrome c folding pathway: evidence for a sequential stabilization mechanism. J. Mol. Biol. 359, 1410–1419. (10.1016/j.jmb.2006.04.035) 16690080

[18] Zhou H Zhou Y 2002 Folding rate prediction using total contact distance. Biophys. J. 82, 458–463. (10.1016/S0006-3495(02)75410-6) 11751332 PMC1302485

[19] Illingworth CJ Scott PD Parkes KE Snell CR Campbell MP Reynolds CA 2010 Connectivity and binding-site recognition: applications relevant to drug design. J. Comput. Chem. 31, 2677–2688. (10.1002/jcc.21561) 20839295

[20] Sobolev V Sorokine A Prilusky J Abola EE Edelman M 1999 Automated analysis of interatomic contacts in proteins. Bioinformatics 15, 327–332. (10.1093/bioinformatics/15.4.327) 10320401

[21] Hobohm U Scharf M Schneider R Sander C 1992 Selection of representative protein data sets. Protein Sci. 1, 409–417. (10.1002/pro.5560010313) 1304348 PMC2142204

[22] Hobohm U Sander C 1994 Enlarged representative set of protein structures. Protein Sci. 3, 522–524. (10.1002/pro.5560030317) 8019422 PMC2142698

[23] Yang LW Bahar I 2005 Coupling between catalytic site and collective dynamics: a requirement for mechanochemical activity of enzymes. Structure 13, 893–904. (10.1016/j.str.2005.03.015) 15939021 PMC1489920

[24] Sacquin-Mora S Lavery R 2006 Investigating the local flexibility of functional residues in hemoproteins. Biophys. J. 90, 2706–2717. (10.1529/biophysj.105.074997) 16428284 PMC1414562

[25] Sacquin-Mora S Laforet E Lavery R 2007 Locating the active sites of enzymes using mechanical properties. Proteins 67, 350–359. (10.1002/prot.21353) 17311346

[26] Plaxco KW Simons KT Baker D 1998 Contact order, transition state placement and the refolding rates of single domain proteins. J. Mol. Biol. 277, 985–994. (10.1006/jmbi.1998.1645) 9545386

[27] Ivankov DN Garbuzynskiy SO Alm E Plaxco KW Baker D Finkelstein AV 2003 Contact order revisited: influence of protein size on the folding rate. Protein Sci. 12, 2057–2062. (10.1110/ps.0302503) 12931003 PMC2324001

[28] Gromiha MM Selvaraj S 2001 Comparison between long-range interactions and contact order in determining the folding rate of two-state proteins: application of long-range order to folding rate prediction. J. Mol. Biol. 310, 27–32. (10.1006/jmbi.2001.4775) 11419934

[29] Harihar B Selvaraj S 2009 Refinement of the long-range order parameter in predicting folding rates of two-state proteins. Biopolymers 91, 928–935. (10.1002/bip.21281) 19603493

[30] DeSancho D Munoz V 2011 Integrated prediction of protein folding and unfolding rates from only size and structural class. Phys. Chem. Chem. Phys. 13, 17 030–17 043. 10.1039/c1cp20402e 21670826

[31] Ouyang Z Liang J 2008 Predicting protein folding rates from geometric contact and amino acid sequence. Protein Sci. 17, 1256–1263. (10.1110/ps.034660.108) 18434498 PMC2441995

[32] Maxwell KL 2005 Protein folding: defining a ‘standard’ set of experimental conditions and a preliminary kinetic data set of two-state proteins. Protein Sci. 14, 602–616. (10.1110/ps.041205405) 15689503 PMC2279278

[33] Zou T Ozkan SB 2011 Local and non-local native topologies reveal the underlying folding landscape of proteins. Phys. Biol. 8, 066011 (10.1088/1478-3975/8/6/066011) 22146659

[34] White SH Wimley WC 1999 Membrane protein folding and stability: physical principles. Annu. Rev. Biophys. Biomol. Struct. 28, 319–365. (10.1146/annurev.biophys.28.1.319) 10410805

[35] Galzitskaya OV Garbuzynskiy SO Ivankov DN Finkelstein AV 2003 Chain length is the main determinant of the folding rate for proteins with three-state folding kinetics. Proteins 51, 162–166. (10.1002/prot.10343) 12660985

[36] Paci E Lindorff-Larsen K Dobson CM Karplus M Vendruscolo M 2005 Transition state contact orders correlate with protein folding rates. J. Mol. Biol. 352, 495–500. (10.1016/j.jmb.2005.06.081) 16120445

[37] Vendruscolo M Dokholyan NV Paci E Karplus M 2002 Small-world view of the amino acids that play a key role in protein folding. Phys. Rev. E. 65, 061910 (10.1103/PhysRevE.65.061910) 12188762

[38] Vendruscolo M Paci E Dobson CM Karplus M 2001 Three key residues form a critical contact network in a protein folding transition state. Nature 409, 641–645. (10.1038/35054591) 11214326

[39] Rustad M Ghosh K 2012 Why and how does native topology dictate the folding speed of a protein? J. Chem. Phys. 137, 205104 (10.1063/1.4767567) 23206039

[40] Makarov DE Keller CA Plaxco KW Metiu H 2002 How the folding rate constant of simple, single-domain proteins depends on the number of native contacts. Proc. Natl Acad. Sci. USA 99, 3535–3539. (10.1073/pnas.052713599) 11904417 PMC122558

[41] Plaxco KW Simons KT Ruczinski I Baker D 2000 Topology, stability, sequence, and length: defining the determinants of two-state protein folding kinetics. Biochemistry 39, 11 177–11 183. (10.1021/bi000200n) 10985762

[42] Northey JG Di Nardo AA Davidson AR 2002 Hydrophobic core packing in the SH3 domain folding transition state. Nat. Struct. Biol. 9, 126–130. (10.1038/nsb748) 11786916

[43] Piana S Lindorff-Larsen K Shaw DE 2011 How robust are protein folding simulations with respect to force field parameterization? Biophys. J. 100, L47–L49. (10.1016/j.bpj.2011.03.051) 21539772 PMC3149239

[44] Warne T Serrano-Vega MJ Baker JG Moukhametzianov R Edwards PC Henderson R Leslie AG Tate CG Schertler GF 2008 Structure of a beta1-adrenergic G-protein-coupled receptor. Nature 454, 486–491. (10.1038/nature07101) 18594507 PMC2923055

[45] Kobayashi H Ogawa K Yao R Lichtarge O Bouvier M 2009 Functional rescue of beta-adrenoceptor dimerization and trafficking by pharmacological chaperones. Traffic 10, 1019–1033. (10.1111/j.1600-0854.2009.00932.x) 19515093 PMC2755524

[46] McCallister EL Alm E Baker D 2000 Critical role of beta-hairpin formation in protein G folding. Nat. Struct. Biol. 7, 669–673. (10.1038/77971) 10932252

[47] Kim DE Fisher C Baker D 2000 A breakdown of symmetry in the folding transition state of protein L. J. Mol. Biol. 298, 971–984. (10.1006/jmbi.2000.3701) 10801362

[48] Northey JG Maxwell KL Davidson AR 2002 Protein folding kinetics beyond the phi value: using multiple amino acid substitutions to investigate the structure of the SH3 domain folding transition state. J. Mol. Biol. 320, 389–402. (10.1016/S0022-2836(02)00445-X) 12079394

[49] Hubner IA Shimada J Shakhnovich EI 2004 Commitment and nucleation in the protein G transition state. J. Mol. Biol. 336, 745–761. (10.1016/j.jmb.2003.12.032) 15095985

[50] Itzhaki LS Otzen DE Fersht AR 1995 The structure of the transition state for folding of chymotrypsin inhibitor 2 analysed by protein engineering methods: evidence for a nucleation–condensation mechanism for protein folding. J. Mol. Biol. 254, 260–288. (10.1006/jmbi.1995.0616) 7490748

[51] Otzen DE Itzhaki LS elMasry NF Jackson SE Fersht AR 1994 Structure of the transition state for the folding/unfolding of the barley chymotrypsin inhibitor 2 and its implications for mechanisms of protein folding. Proc. Natl Acad. Sci. USA 91, 10 422–10 425. (10.1073/pnas.91.22.10422) PMC45032 7937967

[52] Leszczynski JF Rose GD 1986 Loops in globular proteins: a novel category of secondary structure. Science 234, 849–855. (10.1126/science.3775366) 3775366

[53] Ghosh K Dill KA 2009 Theory for protein folding cooperativity: helix bundles. J. Am. Chem. Soc. 131, 2306–2312. (10.1021/ja808136x) 19170581 PMC2654554

[54] Shibata Y White JF Serrano-Vega MJ Magnani F Aloia AL Grisshammer R Tate CG 2009 Thermostabilization of the neurotensin receptor NTS1. J. Mol. Biol. 390, 262–277. (10.1016/j.jmb.2009.04.068) 19422831 PMC2696590

[55] Serrano-Vega MJ Magnani F Shibata Y Tate CG 2008 Conformational thermostabilization of the beta1-adrenergic receptor in a detergent-resistant form. Proc. Natl Acad. Sci USA 105, 877–882. (10.1073/pnas.0711253105) 18192400 PMC2242685

[56] Schlinkmann KM Honegger A Tureci E Robison KE Lipovsek D Pluckthun A 2012 Critical features for biosynthesis, stability, and functionality of a G protein-coupled receptor uncovered by all-versus-all mutations. Proc. Natl Acad. Sci. USA 109, 9810–9815. (10.1073/pnas.1202107109) 22665811 PMC3382542

